# Emergence of *IGH::CCND1* rearrangement and mutations in *TP53*, *BTK*, and *BCL2* associated with therapy resistance in chronic lymphocytic leukemia

**DOI:** 10.1002/jha2.1024

**Published:** 2024-10-08

**Authors:** Qing Wei, Hong Fang, James M. Jing, Roman Segura‐Rivera, L. Jeffrey Medeiros, Wei Wang

**Affiliations:** ^1^ Department of Hematopathology The University of Texas MD Anderson Cancer Center Houston Texas USA; ^2^ Department of Abdominal Imaging The University of Texas MD Anderson Cancer Center Houston Texas USA

**Keywords:** CLL, IGH:CCND1 rearrangement, mutations, therapy‐resistance

## Abstract

Chronic lymphocytic leukemia (CLL) is an indolent low‐grade B‐cell neoplasm that generally responds well to treatment. Rarely, CLL cases exhibit an IGH::CCND1 rearrangement, presenting a diagnostic challenge in distinguishing between clonal evolution within CLL and an independent mantle cell lymphoma. We report a case of CLL with the emergence of an IGH::CCND1 rearrangement and mutations associated with treatment resistance, including TP53, BTK, and BCL2, during disease progression. Immunophenotypic, cytogenetic, and molecular analyses support that these alterations are secondary events within the CLL clone. This case demonstrates dynamic clonal selection and expansion in response to treatments, which, in turn, contributes to treatment resistance.

## 1 INTRODUCTION

Chronic lymphocytic leukemia (CLL) is an indolent low‐grade B‐cell neoplasm that often responds to therapy with a good prognosis. We present a patient with CLL who became resistant to multiple therapeutic regimens, which were associated with the emergence of *IGH::CCND1* rearrangement and sequential acquisition of mutations in *TP53*, *BTK*, and *BCL2* during disease progression. The emergence of *IGH::CCND1* presents a diagnostic dilemma, raising questions about whether this rearrangement is a secondary event within the CLL clone or an independent process diagnostic for mantle cell lymphoma. The emergence of *TP53*, *BTK*, and *BCL2* mutations occurred after standard chemoimmunotherapy, a tyrosine kinase inhibitor, and BCL‐2 inhibitor treatment respectively, indicating dynamic clonal selection and expansion in response to treatments that in turn contributed to treatment resistance.

## 2 CASE REPORT

In 2004, a 42‐year‐old woman was diagnosed with IGHV‐unmutated CLL. Fluorescence in situ hybridization (FISH) analysis was negative for *TP53* deletion. Following three years of observation, the patient's disease progressed with lymphocytosis trending up. She was treated with six cycles of fludarabine, cyclophosphamide, and rituximab and achieved complete remission. In 2013, the patient experienced her first relapse and underwent treatment with rituximab and cyclophosphamide for six cycles. In 2015, the patient had a second relapse and next‐generation sequencing (NGS) showed three *TP53* mutations, all with a low variant allele frequency of less than 5%, including p.R248W, R249S, and p.R342*. She received treatment including rituximab and ibrutinib, with ibrutinib as maintenance therapy.

**FIGURE 1:**
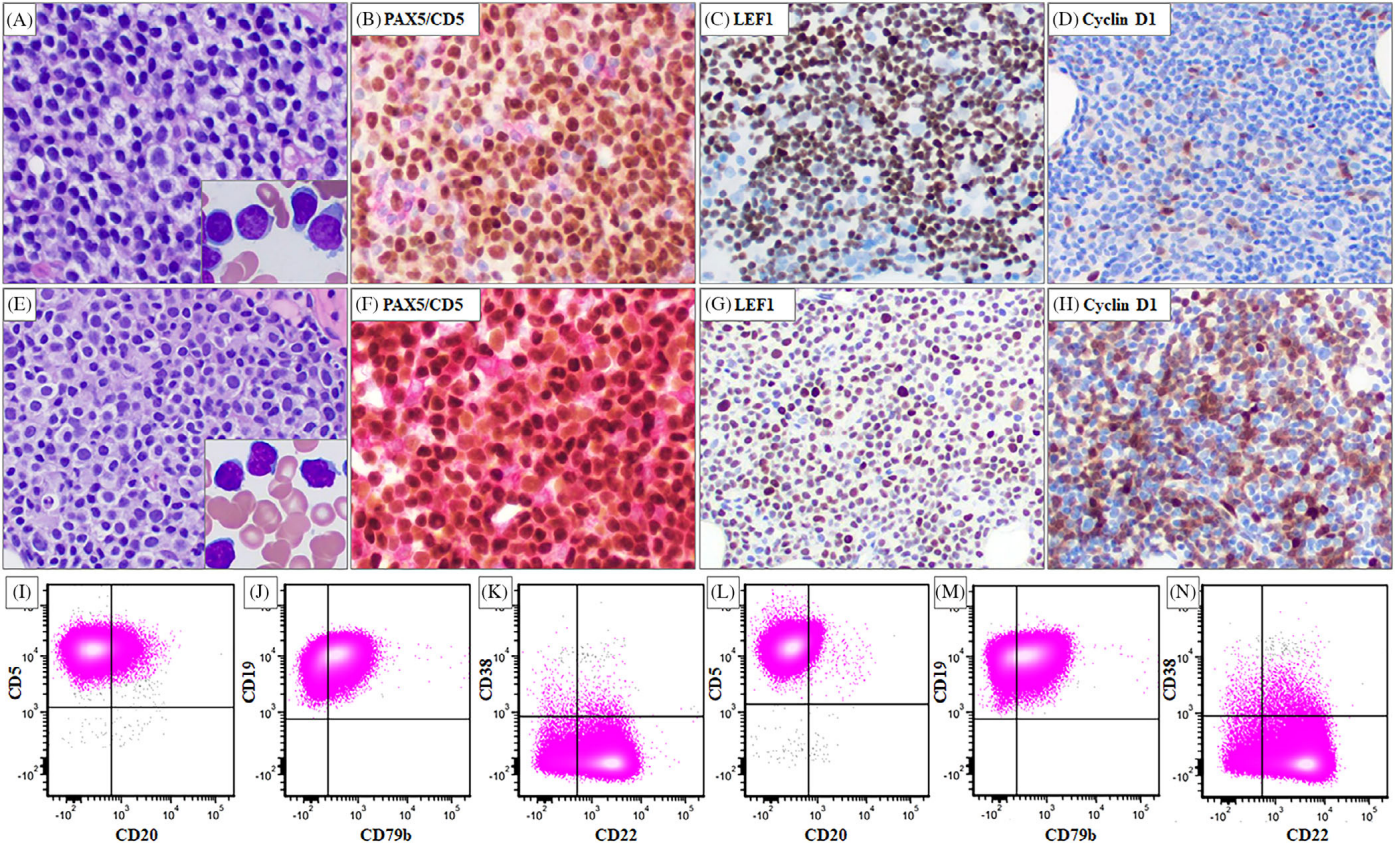
Comparison of morphologic and immunophenotypic findings in bone marrow samples before and after emergence of *IGH::CCND1* rearrangement. Panels (A–D, I–K) Findings before the emergence of *IGH::CCND1* rearrangement. Panels (E–H, L–N) Findings after the emergence of *IGH::CCND1* rearrangement. Lymphoma cells in both bone marrow samples exhibit similar morphology (A, E, inset, bone marrow smears) and are positive for CD5 and PAX5 (B, F, dual staining). LEF1 expression is also positive in both samples (C, G). Cyclin D1 expression is observed in rare, scattered cells in the first bone marrow sample (D), whereas most lymphoma cells in the second bone marrow sample are positive for Cyclin D1 (H). Immunophenotypic analysis by flow cytometry before (I–K) and after (L–N) the emergence of *IGH::CCND1* shows that lymphoma cells in both samples are positive for CD22 and weakly positive for CD79b, while CD20 is largely negative.

In 2021, the patient presented with a white blood cell count of 56.1 k/uL with 96% lymphocytes, lymphadenopathy, and B symptoms. Bone marrow aspiration and biopsy revealed CLL composed of predominantly small mature lymphocytes with scattered large cells, some with distinct nucleoli. The biopsy specimen showed a nodular and interstitial pattern, representing 50%–60% of bone marrow cellularity (Figure 1A). Flow cytometric analysis showed a typical CLL immunophenotype (Figure 1I–K). Immunochemical analysis on the biopsy specimen showed that the neoplastic cells were positive for CD5, PAX5, and LEF1. Cyclin D1 is largely negative with rare positive ones (Figure 1B–D). Conventional cytogenetic analysis showed a complex karyotype and FISH analysis showed D13S319 and *TP53* loss (Table 1). NGS detected a *TP53* mutation (p.R342*) that was identical to the mutation detected in the 2015 specimen. In addition, mutations in *BTK* and *RPS15* were detected (Table 1). The patient started treatment with venetoclax and ibrutinib and showed disease progression while on therapy. The treatment was then switched to obinutuzumab and venetoclax for six cycles, followed by venetoclax maintenance.

**TABLE 1:** The comparison of karyotype, FISH, NGS, and IGH rearrangement between two bone marrow specimens without and with *IGH::CCND1* rearrangement.

	Karyotype	FISH	NGS	IGH rearrangement
2021	43–44, XX, del(3)(q27), add(8)(p11.2), ‐9, add(9)(p21), add(10)(q26), del(13)(q12q22), ‐15, ‐ 17, add(18)(q23), +2∼3mar[cp4]/46, XX[16]	D13S319 78% loss *TP53* 78% loss	*BTK* p. C481S, 35% *RPS15* p.S139F 36% *TP53* p.R342* 32%	Both are monoclonal with the same size of PCR products using FR1, FR2, and FR3 primer sets.
2023	43–44, XX, inv(4)(p14q21), add(6)(p23), ‐9, add(10)(q26), **t(11;14)(q13;q32)**, del(13)(q12q22), ‐15, ‐17, ‐18, add(18)(q23), +1∼2mar [cp16]/46, XX[4]	D13S319 87% loss *TP53* 88.5% loss ***IGH::CCND1***	*BTK* p.C481S 38% *RPS15* p.S139F 40% *TP53* p.R342* 57% ***BCL2* p.A113G 6%** ***BCL2* p.R110_D111insHYRR,7%** ***BCL2* p.G101V 7%** ***BCL2* p. D103E 10%**	

*Note*: The major differences are highlighted in bold. Under karyotype, cytogenetic abnormalities shared by the two bone marrow specimens are underlined.

A subsequent bone marrow aspiration and biopsy in 2023 showed CLL involving 60% of the bone marrow cellularity. When compared with the 2021 bone marrow specimen, similar morphologic (Figure 1E) and immunophenotypic findings (Figure 1L–N) were identified. Immunohistochemical analysis showed that the neoplastic cells were positive for CD5, PAX5, and LEF1, which was similar to that identified in the 2021 specimen (Figure 1F,G). However, lymphoma cells were positive for cyclin D1 (Figure 1H) and negative for SOX11 (not shown). Conventional chromosomal analysis revealed a complex karyotype associated with a newly emerged t(11;14)(q13;q32) (Table 1). FISH analysis confirmed *IGH::CCND1* rearrangement. NGS showed persistent mutations in *BTK*, *RPS15*, and *TP53* that were present in the 2021 specimen. In addition, four new mutations in *BCL2* were identified (Table 1).

The detection of the *IGH::CCND1* translocation in the 2023 bone marrow specimen raises questions about whether the patient developed a second lymphoma—mantle cell lymphoma (MCL) or this rearrangement is a manifestation of clonal evolution within CLL. In this case, strong evidence of clonal relationship between the 2021 and 2023 bone marrow specimens supports the latter: 1) The lymphoma cells showed a persistent CLL immunophenotype and LEF1 positivity (Figure 1) in both samples; 2) Cytogenetic studies showed overlapping abnormalities such as add(10)(q26), del(13), ‐15, ‐17, and add(18)(q23) by karyotype and D13S319 and *TP53* loss by FISH (Table 1); 3) NGS detected identical mutations in *BTK*, *RPS15*, and *TP53*. The additional mutations in *BCL2* (each VAF ≤ 10%) indicated emerging subclones (Table 1); and 4) Immunoglobulin clonality studies showed an identical clonal IGH rearrangement in the 2021 and 2023 bone marrow specimens (data not shown) confirming their clonal relationship.

## 3 DISCUSSION

Cases of composite lymphoma composed of CLL and MCL have been documented, typically characterized by two separate processes with two independent clones [1]. Our case, along with rare previously reported cases [2, 3, 4], demonstrates that *IGH::CCND1* translocation can be an acquired event deriving from the CLL clone during disease progression. Investigating clonal relationship through cytogenetic and molecular studies is crucial in these scenarios, as composite lymphoma is usually clonally unrelated whereas CLL with secondary acquisition of t(11;14)(q13;q32)/*IGH::CCND1* implies clonal relatedness and disease progression. Furthermore, our case underscores the value of NGS in elucidating resistance mechanisms and guiding subsequent therapy. We observed *TP53* clonal expansion following rituximab and cyclophosphamide therapy, aligning with other studies indicating expansion of *TP53* clonal variants associated with fludarabine, cyclophosphamide, and rituximab regimens [5]. The presence of del(17p)/*TP53* mutation is associated with resistance to chemotherapy, warranting targeted therapy [6]. Our patient switched to targeted therapy composed of rituximab and ibrutinib. Subsequently, the emergence of *BTK* p.C481S mutation, a known genetic driver of resistance to ibrutinib [7], occurred after ibrutinib therapy, which prompted a switch to treatment with the BCL2 inhibitor venetoclax. Identification of *BCL2* mutations in the 2023 bone marrow specimen suggests the emergence of several potentially resistant subclones following venetoclax treatment [8].

In summary, our case demonstrates that *IGH::CCND1* rearrangement is a rare genetic event that can emerge within a CLL clone during disease progression. This case also illustrates how sequential mutations, which may respond to various treatments, can contribute to treatment resistance in a CLL patient.

## AUTHOR CONTRIBUTIONS

Wei Wang designed the research. Qing Wei, Hong Fang, James M. Jing, Roman Segura‐Rivera, L. Jeffrey Medeiros, and Wei Wang wrote and approved the manuscript.

## CONFLICT OF INTEREST STATEMENT

The authors declare no conflict of interest.

## FUNDING INFORMATION

The authors received no specific funding for this work.

## ETHICS STATEMENT

The study was performed in accordance with the principles of the Declaration of Helsinki and the institutional guidelines.

## PATIENT CONSENT STATEMENT

The authors have confirmed patient consent statement is not needed for this submission.

## CLINICAL TRIAL REGISTRATION

The authors have confirmed clinical trial registration is not needed for this submission.

## Data Availability

No datasets were generated in this study.
